# Molecular cloning, expression, and characterization of four novel thermo-alkaliphilic enzymes retrieved from a metagenomic library

**DOI:** 10.1186/s13068-017-0808-y

**Published:** 2017-06-02

**Authors:** Mukil Maruthamuthu, Jan Dirk van Elsas

**Affiliations:** Department of Microbial Ecology, Groningen Institute for Evolutionary Life Sciences, University of Groningen, Nijenborgh 7, 9747AG Groningen, The Netherlands

**Keywords:** Recombinant glycoside hydrolases, Thermo-alkaliphilic enzymes, Galactosidase, Biomass pretreatment, Biotechnological application

## Abstract

**Background:**

Enzyme discovery is a promising approach to aid in the deconstruction of recalcitrant plant biomass in an industrial process. Novel enzymes can be readily discovered by applying metagenomics on whole microbiomes. Our goal was to select, examine, and characterize eight novel glycoside hydrolases that were previously detected in metagenomic libraries, to serve biotechnological applications with high performance.

**Results:**

Here, eight glycosyl hydrolase family candidate genes were selected from metagenomes of wheat straw-degrading microbial consortia using molecular cloning and subsequent gene expression studies in *Escherichia coli.* Four of the eight enzymes had significant activities on either *p*NP-β-d-galactopyranoside, *p*NP-β-d-xylopyranoside, *p*NP-α-l-arabinopyranoside or *p*NP-α-d-glucopyranoside. These proteins, denoted as proteins 1, 2, 5 and 6, were his-tag purified and their nature and activities further characterized using molecular and activity screens with the *p*NP-labeled substrates. Proteins 1 and 2 showed high homologies with (1) a β-galactosidase (74%) and (2) a β-xylosidase (84%), whereas the remaining two (5 and 6) were homologous with proteins reported as a diguanylate cyclase and an aquaporin, respectively. The β-galactosidase- and β-xylosidase-like proteins 1 and 2 were confirmed as being responsible for previously found thermo-alkaliphilic glycosidase activities of extracts of *E. coli* carrying the respective source fosmids. Remarkably, the β-xylosidase-like protein 2 showed activities with both *p*NP-Xyl and *p*NP-Ara in the temperature range 40–50 °C and pH range 8.0–10.0. Moreover, proteins 5 and 6 showed thermotolerant α-glucosidase activity at pH 10.0. In silico structure prediction of protein 5 revealed the presence of a potential “GGDEF” catalytic site, encoding α-glucosidase activity, whereas that of protein 6 showed a “GDSL” site, encoding a ‘new family’ α-glucosidase activity.

**Conclusion:**

Using a rational screening approach, we identified and characterized four thermo-alkaliphilic glycosyl hydrolases that have the potential to serve as constituents of enzyme cocktails that produce sugars from lignocellulosic plant remains.

**Electronic supplementary material:**

The online version of this article (doi:10.1186/s13068-017-0808-y) contains supplementary material, which is available to authorized users.

## Background

Plant biomass is considered to represent a sustainable source of sugars for biofuel production via fermentation. In this biomass, lignocellulosic material is the key source of ‘renewable’ energy [1]. Lignocellulose consists of the major compounds cellulose (40–50%) and hemicellulose (25–30%), next to lignin [2]. Hemicellulose is composed of different pentose (xylose, arabinose) and hexose (mannose, galactose, glucose) sugars that are linked by α- and/or β-glycosidic bonds [3]. All of these sugars are widely used in biotechnology to produce bio-based materials, such as biofuels, plastics and other chemicals [4, 5]. In particular, hemicellulases are key to the degradation of plant biomass; the hemicellulose fraction of the plant biomass represents a rich source of d-xylose, which is considered to constitute a key sugar for further biotechnological approaches. Thus, in the light of the currently still imperfect substrate unlocking approaches [6], a great interest has arisen to enhance the industrial enzyme-mediated lignocellulose hydrolysis methods [7], with hemicellulose as a prime target.

Different sets of hydrolytic enzymes (glycosyl hydrolases) are likely required for the complete deconstruction of hemicellulose compounds in order to obtain a mixture of sugars. In nature, communities of microorganisms, which include fungi and bacteria, often produce mixtures of glycosyl hydrolases (GHases) that complete the lignocellulose breakdown processes [8, 9]. Moreover, to increase the efficiency of industrial hydrolysis for lignocellulose breakdown, multi-species microbial consortia play vital roles [10–12]. However, despite large research efforts over the past decade, our limited understanding of how the glycosyl hydrolases and their associated enzymes and/or proteins function together to break down lignocellulosic materials remains a key limitation for many applications [13].

In a previous study, we constructed fosmid libraries from two wheat straw-degrading microbial consortia, which were subsequently screened for the presence of genes for (hemi)cellulose-degrading enzymes using a multi-substrate approach [14]. In this endeavor, we screened for the presence of genes encoding 12 different GH family proteins (using CAZy database annotation), which were considered to possess the desired enzymatic activities, i.e., β-galactosidase, β-xylosidase and α-glucosidase. Heat- and alkali-tolerant enzymatic activities were found with extracts produced from four *E. coli* fosmid clones, denoted NT2-2, T4-1, T5-5 and NT18-17 [14]. The first three clones were identified as containing genes encoding proteins with β-galactosidase and β-xylosidase activities. On the other hand, clone NT18-17 presumably carried a gene for a protein with α-glucosidase activity, next to those for other glycoside hydrolase family enzymes, as predicted by CAZy database annotation. One more fosmid clone, 10BT, revealed enzymatic activity with mixtures of four substrates; in it, genes for proteins of families GH39 and GH53 were identified. However, the work with these five fosmid clones, into each of which up to 35 kb of metagenomic DNA (encompassing up to 30 genes), was cloned, precluded the precise determination of the exact function of each of the predicted proteins.

In the current study, we selected eight genes from the aforementioned five fosmid clones, of which three were predicted to produce enzymes with novel thermo-alkaliphilic activity. The genes were subcloned in the pET28b(+) expression vector and (over)expressed in *E. coli*, after which the gene products were purified and biochemically characterized. The study explored, confirmed and refined the hypothesis that the selected proteins indeed have the thermo-alkaliphilic activities predicted from our previous study [14]. The characterization and in vitro studies identified two new glycoside hydrolase family enzymes with α-glucosidase activities.

## Methods

### Cloning system

Cloning vector pET28b(+) (Novagen, Amsterdam, The Netherlands) was used for the expression of the selected genes. *Escherichia coli* JM109 competent cells (Promega, Leiden, The Netherlands), as well as BL21(DE3) and Origami2 (DE3) pLysS cells (Novagen, Amsterdam, The Netherlands) were used as host strains for cloning and expression studies. Restriction enzymes (*Eco*R1, *Bam*H1, *Hin*dIII, and *Xho*1) and T4-DNA ligase were purchased from Fermentas (Amsterdam, The Netherlands) and used in accordance with the manufacturer’s instructions.

### Extraction of DNA and molecular cloning into expression plasmids

**Fig. 1:**
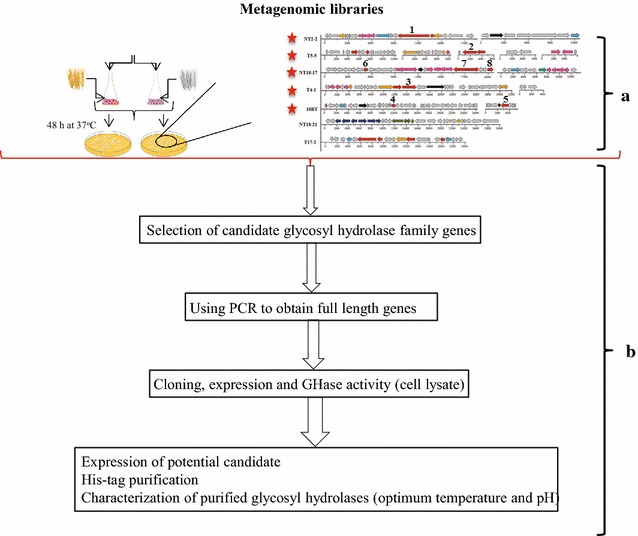
Candidate gene selection and cloning strategy used in this study. **a.** Selected candidate genes from functional screening of fosmid libraries [14]. **b.** Experimental setup

Selected *E. coli* EPI 300 fosmid clones NT2-2, T4-1, T5-5, NT18-17 and 10BT (Fig. 1) were cultured in 4 ml of Luria Broth (LB) supplemented with 12.5 μl/ml chloramphenicol (Cm; Sigma-Aldrich Chemie B. V, Zwijndrecht, The Netherlands). Then, fosmid DNA was extracted as described [14]. PCR primers were designed in regions outside of each gene, adding specific restriction sites to their 5′-ends (Table 1). Thus, full-length genes were generated from the clones by each PCR [Initial denaturation at 98 °C for 30 s followed by 35 cycles of 10 s at 98 °C, 30 s at 64 °C, and 1.5 min at 72 °C, with a final extension step of 72 °C (for 10 min)]. The PCR products were digested with selected restriction enzymes and then analyzed on 1% agarose gels. All patterns were in conformity with the predicted ones (Table 1). Then, full PCR products were run on gel and recovered from it using the Zymoclean™ Large Fragment DNA recovery kit (Zymo Research, Irvine, USA). Following recovery and purification, each DNA fragment was then ligated into expression vector pET28b(+), which was followed by transformation of *Escherichia coli* JM109 competent cells (Promega. Leiden, The Netherlands).

**Table 1:** List of selected genes, PCR primers, and predicted size

Fosmid name	Predicted enzyme (Gene encoding)	Gene no.	Primers	Restriction enzymes	Size (bp)	Approx. size (kDa)	Closet hit (identity/coverage)
NT2-2	β-Galactosidase	1	FP-TACgaattcACGGCATCAGCGCAATAGTC RP-AGggatccCGCTTCGCAACTGAGGTTAC	*Eco*RI *Bam*HI	3146	116	*Enterobacter hormaechei* (74/81%)
T5-5	β-Xylosidase	2	FP-GCGaagcttGTGTAGTCTTTGCCCATCTC RP-CAGggatccCGCTTCGCAACTGAGGTTAC	*Hin*dIII *Bam*HI	2410	85	*Enterobacter mori* (83/90%)
T4-1	β-Xylosidase	3	FP-GCGgaattcCACCAGATTCAGCCCTACAG RP-CAGaagcttTGTAGTCTTTGCCCATCTCC	*Eco*RI *Hin*dIII	2439	86	*Enterobacter mori* (83/90%)
10BT	Transcription regulator	4	FP-GCGggatccGCACAACAAGATGGCTTTAC RP-CAGctcgagTAGTCGCATTTTGACGGAAC	*Bam*HI *Xho*I	627	22	*Klebsiella oxytoca* (76/87%)
	Inner membrane protein	5	FP-GCGaagcttGTGCAAAGTATCGCTTTAAC RP-CAGggatccAGCAGCATGAATAAGGACAC	*Hin*dIII *Bam*HI	1262	45	*Enterobacter cloacae* (79/88%)
NT18-17	Aquaporin	6	FP-GCGaagcttGGGATCGGCTAGATCTTGAG RP-CAGggatccCAAGCGTCATGAATAAACTG	*Hin*dIII *Bam*HI	673	22	*Hyphomonas neptunium* (65/80%)
	Beta-hexosaminidase	7	FP-GCGggatccTTTGCGGAGCCTACTCCTTC RP-CAGaagcttTTACAATGGCATCTGATTGG	*Bam*HI *Hin*dIII	1944	70	*Rhizobium leguminosarum* 3841 (50/64%)
	Hypothetical protein	8	FP-GCGggatccCCGGATACTCCACACAAAGG RP-CAGctcgagTTATCGCAAGTCGATCAAGG	*Bam*HI *Xho*I	1134	37	*Hyphomicrobium denitrificans* (56/71%)

### Verification of inserts, and expression and purification of target proteins

**Fig. 2:**
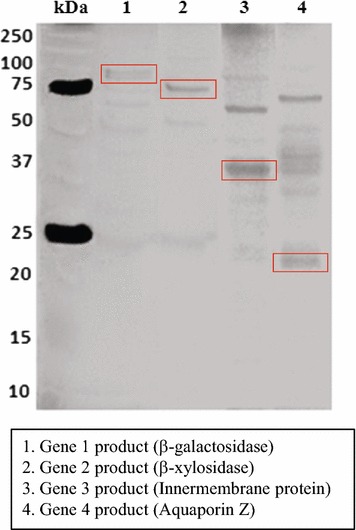
Analysis of His-tag purified proteins. Four selected proteins were checked using 12% SDS-PAGE

All transformations were successful, and so eight recombinant constructs were produced. The success of cloning was further confirmed by colony PCR with the respective primers (Table 1), yielding single amplicons of the expected sizes for all genes (Table 1). This was followed by single and double restriction of the constructs with the relevant enzymes (Table 1). Thus, *Eco*R1/*Bam*H1 was used for gene 1 (3146 bp); *Hin*dIII/*Bam*H1 for gene 2 (2.4 kb); *Eco*R1/*Hin*dIII for gene 3 (2.4 kb); *Bam*H1/*Xho*1 for gene 4 and gene 8 (size of 624 bp and 1134 bp, respectively), and *Hin*dIII/*Bam*H1 for gene 5 (1.2 kb), gene 6 (673 bp), and gene 7 (2.0 kb). Clones carrying the selected eight genes were then selected and purified, after which they were used to inoculate 4ml LB tubes supplemented with 50 μg/ml of kanamycin (to select for the maintenance of the plasmid with insert). Tubes were incubated at 37 °C (shaking at 220 rpm) for 16–19 h. Following incubation, plasmid extraction was carried out using the QIAprep Spin Miniprep Kit (Qiagen, The Netherlands). All recombinant plasmids had the expected inserts; they were further checked by digestion with the aforementioned enzymes and enzyme combinations. Then, the full plasmids were introduced into *E. coli* strains BL21(DE3) and Origami2 (DE3) pLysS (Novagen, Amsterdam, The Netherlands) competent cells, via transformation. These two strains facilitate the testing of the expression of the cloned genes. Selected transformants were purified and the presence of the correct inserts verified. They were then grown in kanamycin (50 μg/ml)-supplemented 2X-PY medium (2 ml; 16 g Bacto-tryptone, 10 g yeast extract, 10 g NaCl.H_2_O/1, pH 8.0) at 37 °C (220 rpm, overnight). A fresh (200 ml) 2xPY flask was then inoculated, establishing an initial OD_600_ of 0.05, after which the culture was grown at 37 °C (shaking, 220 rpm) to an OD_600_ of 0.5–0.6. Afterwards, the culture was incubated for 1 h at 18 °C (shaking, 220 rpm), after which gene expression was induced by adding isopropyl β-d-1-thiogalactopyranoside (IPTG) at 0.5 M. Then, the culture was further incubated at 18 °C for 16–19 h, after which cells were harvested at 4000×*g* (4 °C, 15 min). The pellets were resuspended in 5 ml of lysis buffer (50 mM HEPES, pH 8.0, 300 mM NaCl, 50 µl 1 M DTT (1,4-Dithiothreitol), 1 protease inhibitor mini tablet (Roche, Sigma-Aldrich Chemie B. V, Zwijndrecht, The Netherlands) and the mixtures kept on ice for 15 min. Then, cells were disrupted using sonification with the following parameters: 40 cycles—6 s on/15 s off—amplitude 6–10 µm. After this treatment, the resulting cell lysates were centrifuged at 15,000*g* for 15 min at 4 °C. The supernatants were removed and stored, and 10 µl was checked with 12% SDS-PAGE (sodium dodecyl sulfate polyacrylamide gel electrophoresis), followed by staining with the Pierce™ 6xHis protein tag stain reagent set (Thermo Fisher Scientific, Waltham, USA). The preparation was then heated to 60 °C for 15–20 min and centrifuged at 15,000×*g* to remove insoluble debris. Purification of his-tagged proteins from the crude extracts was then carried out by gravity flow chromatography through agarose. Thus, 600 μl of Ni–NTA agarose (Qiagen, Hilden, Germany) was added to 10 ml of lysis buffer. Incubation was for 5 min (shaking, 4 °C), before the mixture was centrifuged for 5 min at 800*g* at 4 °C. The supernatant was discarded and then 10 ml of equilibration buffer (50 mM HEPES and 300 mM NaCl) was added, after which the mixture was incubated as mentioned above. A short spin followed. Then, the crude extract was added to the resin and incubated for 1 h before it was transferred into a gravity flow column and incubated at 4 °C until the resin bed settled down. The cell-free lysates were removed by gravity flow and unbound proteins were washed 3 times with 10 ml of wash buffer (50 mM HEPES, 300 mM NaCl, 20 mM Imidazole). The bound enzyme was eluted with 3 ml of elution buffer (50 mM HEPES, 300 mM NaCl, 400 mM Imidazole). The enzyme samples were concentrated using Amicon ultra-15 centrifugal filter units (Millipore, Amsterdam, The Netherlands) and quantified using the Bradford method (Bradford 1X dye, Biorad, Veenendaal, The Netherlands). The purity was then analyzed by running 12% SDS-PAGE followed by staining with Pierce™ 6xHis protein tag stain reagent Set (Fig. 2).

### Substrate specificity testing

Fifty microliter volumes containing approximately 0.6 μg of enzyme were used for testing enzyme activity on 3, 5 and 10 mM of *p*NP substrate (*p*NP-β-d-galactopyranoside, *p*NP-β-d-xylopyranoside, *p*NP-α-l-arabinopyranoside, and *p*NP-α-d-glucopyranoside) (Sigma-Aldrich Chemie B. V, Zwijndrecht, The Netherlands). Fifty mM of Tris–HCl buffer—pH 7.5 was used in the ratio of 1:1. The reaction mixture was incubated at 40 °C for 0.5–1 h, after which the reactions were deactivated on ice for 10 min. To validate the enzyme activity, the experiments were controlled with (i) water and substrate, (ii) enzyme with water, and (iii) host protein lysate with substrate. The concentration of released *p*NP was determined by measuring the reaction mixture absorbance at 410 nm using a calibration curve, as explained [14]. Effects of temperature and pH on hydrolysis activity for all recombinant enzymes were evaluated. For temperature, we used the range 10–70 °C at pH 7.5. For pH, we used the range 4.0–10.0 at 50 °C, using sodium citrate buffer (pH 4.0 and 5.0), Tris–HCl (pH 7.5, 8.0, and 9.0), and glycine–NaOH buffer (pH 10.0).

Kinetic parameters (*K*_m_ and *V*_max_) for all purified recombinant enzymes (protein 1, 2, 5 and 6) were evaluated by measuring the enzyme activity using 0–10 mM of respective *p*NP as substrate in 50 mM Tris–HCl buffer (pH 7.5) at 40 °C for 15 min. The data were plotted according to the Lineweaver–Burk method to calculate *K*_m_ and *V*_max_ values. For thermal stability assays, the purified enzymes were pre-incubated at 50 °C (according to preliminary data) in the absence of *p*NP substrates. After incubation for different time periods (0, 15, 30, 120 and 180 min), enzymatic activity was measured for each enzyme with specific *p*NP substrate, temperature and pH. The inhibitory effect of different concentrations (0–1.0%) of 5-hydroxymethylfurfural (5-HMF) and furfural was determined by incubating all enzymes (protein 1, 2, 5 and 6) with respective series of *p*NP dissolved in 50 mM Tris–HCl buffer (pH 7.5). For the lignocellulose hydrolysis, the reaction mixtures contained 20 mg of raw wheat straw (RWS) substrate with the enzyme (protein 1, 2, 5, 6 and mixed) treatment, adjusted to 1.5 ml with sodium phosphate buffer (0.1 M, pH 6.0). The experiments were carried out at 50 °C (12–24 h, shaking at 250 rpm). After incubation, the mixtures were centrifuged (12,000×*g* for 15 min at 4 °C), and the supernatants collected. The amount of total reducing sugars in the supernatants was measured by the dinitrosalicylic acid (DNS) colorimetric method.

### Activity of selected enzymes in the presence of different ions

The effects of MgCl_2_, MnCl_2_ (5 and 20 mM), and NaCl (20 and 2000 mM) were assessed by measuring enzyme activities with specific substrates in the presence versus absence of these additives. Given the potential importance for application, we used a range of pH (4.0–10.0) and temperatures (40–50 °C). Statistical comparisons among samples were performed using one-way ANOVA (Tukey’s test) using the software Past3 (http://folk.uio.no/ohammer/past/).

### Amino acid sequence and structure analyses of the gene 5 and 6 products

The products of genes 5 and 6 (proteins 5 and 6) were investigated for homology with proteins in the non-redundant protein database (http://www.ncbi.nlm.nih.gov) using BLAST-P [15]. In addition, protein domains were characterized by searching the protein family database Pfam [16]. The two proteins were initially annotated as (1) a diguanylate cyclase and (2) an aquaporin Z. For each gene, a multiple sequence alignment was constructed using COBALT, a tool that finds a collection of pairwise constraints derived from conserved domain database, protein motif database and sequence similarity, using RPS-BLAST, BLAST-P and PHI-BLAST [17]. In addition, domain analyses were done for function prediction by the conserved domain architecture retrieval tool (CDART) [18].

#### Structure analyses

We used Phyre2 to reconstruct the tertiary structures of proteins 5 and 6 [19]. Next, we generated a list of proteins with structural similarities to the protein models using the Dali server [20]. A three-dimensional model of the best three matches for each protein was retrieved from the Protein Data Bank (PDB) [21]. For protein 5, these matches were: activated response regulators, namely a signaling protein [PDB: 1W25], a transferase [PDB: 2WB4], and a lyase [2V0N] from *Caulobacter vibrioides* (Alphaproteobacteria). For protein 6, the matches were membrane proteins (porins) from *Agrobacterium fabrum* [PDB: 3LLQ] and *Escherichia coli* [PDB: 2O9D] and a transport protein, also from *E. coli* [PDB: 3NKA]. Further manipulations, structural alignments and comparisons between the 3D models of the proteins were completed using PyMOL (http://www.pymol.org).

## Results

### Selection of genes encoding glycosyl hydrolases from five fosmid clones

In our previous study, we successfully screened two metagenomic libraries generated from wheat straw-degrading microbial consortia produced by the dilution-to-stimulation approach for genes encoding (hemi)cellulose-degrading enzymes [14]. In total, we identified 18 genes for proteins belonging to 12 different glycosyl hydrolase (GH) families and another 21 genes for carbohydrate-active enzymes (CBMs, AAs, GTs, and CEs; as evidenced by CAZy database annotation), in seven fosmid clones. Five of these fosmid clones, notably NT2-2, T4-1, T5-5, 10BT and NT18-17, were selected here in order to study their enzyme activities in greater detail and match structure with function. Thus, eight genes, denoted genes 1 through 8, encoding putative GHases, were initially selected in these clones (Table 1). This was done on the basis of homologies with genes for known GH active enzymes, as well as protein sizes. Interestingly, three of the five selected fosmid clones, denoted NT2-2, T5-5 and NT18-17, had been predicted to encode putative thermo-alkaliphilic enzymes [14].

Here, we provide the specifics of the eight selected genes. Gene 1 (fosmid NT2-2) was annotated as a gene for a family GH2 β-galactosidase (EC. 3.2.1.23), with a molecular size of ~116 kDa. Its predicted amino acid sequence revealed 74% identity with a β-galactosidase of *Enterobacter hormaechei* by Blast-P. Genes 2 (fosmid T5-5) and 3 (fosmid T4-1) were both predicted to encode a family GH3 β-xylosidase of ~86 kDa (EC. 3.2.1.37; tracked to *Enterobacter mori* with identities of 84 and 83%, respectively). These two genes revealed 86% identity between them. The remaining five genes, i.e., genes 4 and 5 (source fosmid 10BT) and 6, 7 and 8 (source fosmid NT18-17), were predicted to encode enzymes belonging to CAZy families GH39, GH53, GH27, GH20 and GH58, respectively. Blast-P comparisons showed homologies of the predicted gene products with a suite of different proteins, i.e. a transcriptional regulator of the AraC family (protein 4; GH39; 76% identity; organism *Klebsiella oxytoca*), an inner membrane protein (protein 5; GH53; 79% identity; *Enterobacter cloacae*), an aquaporin Z (protein 6; GH27; 65% identity; *Hyphomonas neptunium*), a beta-hexosaminidase (protein 7; GH20; 50% identity; *Rhizobium leguminosarum*), and a hypothetical protein (protein 8; GH58; 56% identity; *Hyphomicrobium denitrificans*), respectively (Table 1).

### Characterization of cloned genes and gene expression

The eight selected genes (Table 1) were all cloned into the pET28b(+) vector [22], with polyhistidine-tag sequences at both the N and C termini. All genes were, thus, successfully introduced into *E. coli* strains BL21(DE3) and Origami2 (DE3) pLysS. To confirm the fidelity of the cloning, the pET28b(+) plasmids with inserts were extracted from selected clones, per gene, of each of the two strains and the presence of the cloned fragments detected by restriction with specific restriction enzymes followed by gel electrophoresis. The observed sizes of the restriction fragments (data not shown) were consistent with the fragment sizes as predicted from the fosmid-derived gene sequences (Table 1).

Expression of the eight cloned genes was then investigated using extracts of grown cultures of both *E. coli* transformant strains (BL21(DE3) and Origami2 (DE3) pLysS) in 2x-PY medium, following induction by IPTG. Both the cell and the soluble fractions (culture supernatants) were used in the tests. We investigated the effects of IPTG concentration (0.25, 0.5 and 1 mM), temperature (18, 30 and 37 °C; at 200 rpm), and glucose concentration (0.25, 0.5, 0.75 and 1 mM) on the gene expression levels. In strain BL21(DE3), the target proteins were, unfortunately, mostly found in the cell fractions, except the product of gene 2, where ~50% of protein occurred in the soluble fraction (data not shown). In contrast, the use of *E. coli* Origami2 (DE3) pLysS resulted in seven of the eight proteins being present in the soluble fractions, albeit at different levels. Unfortunately, the single remaining protein (product of gene 4) was only detectable in the cell fraction. The collective data revealed that maximal protein production took place, in the selected strain *E. coli* Origami2 (DE3) pLysS, with 0.5 mM IPTG at 18 °C, in the absence of glucose. This was true for all genes (data not shown).

We thus used the total protein-containing lysates of the cultures of *E. coli* Origami2 (DE3) pLysS for analysis of the selected seven gene products, i.e., proteins 1, 2, 3, 5, 6, 7 and 8, on a series of *p*NP-labeled substrates. Indeed, protein 1 revealed high activity on *p*NP-β-d-galactopyranoside, indicating beta-galactosidase-like activity. This was consistent with the activity that had previously been detected in its source fosmid NT2-2. Interestingly, the cultures of *E. coli* with cloned genes 2 and 3 both yielded soluble fractions that revealed dual activities, i.e. transformation of *p*NP-β-d-xylopyranoside (β-xylosidase) and *p*NP-α-l-arabinopyranoside (α-arabinosidase). These genes had been selected from fosmid clones T5-5 and T4-1, respectively, which had previously shown high β-xylosidase activities, but had not shown α-arabinosidase activity. Expectedly, the cultures with cloned gene 4, with predicted β-xylosidase activity, did not yield lysates with any activity on the substrates used. Clearly, the expressed protein of gene 4 was in inclusion bodies, and unfortunately we were unsuccessful in several attempts to recover native forms of it by refolding. In fact, its source fosmid clone 10BT had previously been found to have the gene 4 encoded GH39 family protein linked to β-xylosidase activity [14]. Finally, proteins 5 and 6 [14]) both revealed activities towards *p*NP-α-d-glucopyranoside, and there was no detectable enzymatic activity with any of the other *p*NP substrates (such as *p*NP-β-d-xylopyranoside and *p*NP-α-l-arabinopyranoside). Gene 5 had also been selected from fosmid clone 10BT, which had shown consistent enzyme activity on *p*NP-β-d-xylopyranoside, but not on α-d-glucopyranoside [14]. Gene 6, originating from fosmid clone NT18-17, yielded a product with α-glucosidase activity, which was in line with the predicted activity in its source fosmid. Finally, and against our expectations, the total protein lysates of genes 7 (GH20 family—β-hexosaminidase; EC 3.2.1.52) and 8 (GH58 family—endo-*N*-acetylneuraminidase) did not reveal activities with any of the substrates.

For all further work, we selected the four genes 1, 2, 5 and 6 that had yielded soluble proteins with key promising activities, i.e. β-galactosidase, β-xylosidase and α-glucosidase. Specifically, gene 2 was selected instead of the similar gene 3, because our previous study showed that its mother fosmid clone, T5-5, had yielded extracts in which proteins with thermo-alkaliphilic β-xylosidase activity were present, whereas fosmid clone T4-1 (source for gene 3) showed only slight β-xylosidase activity [14].

### Enzyme sizing and activity

**Fig. 3:**
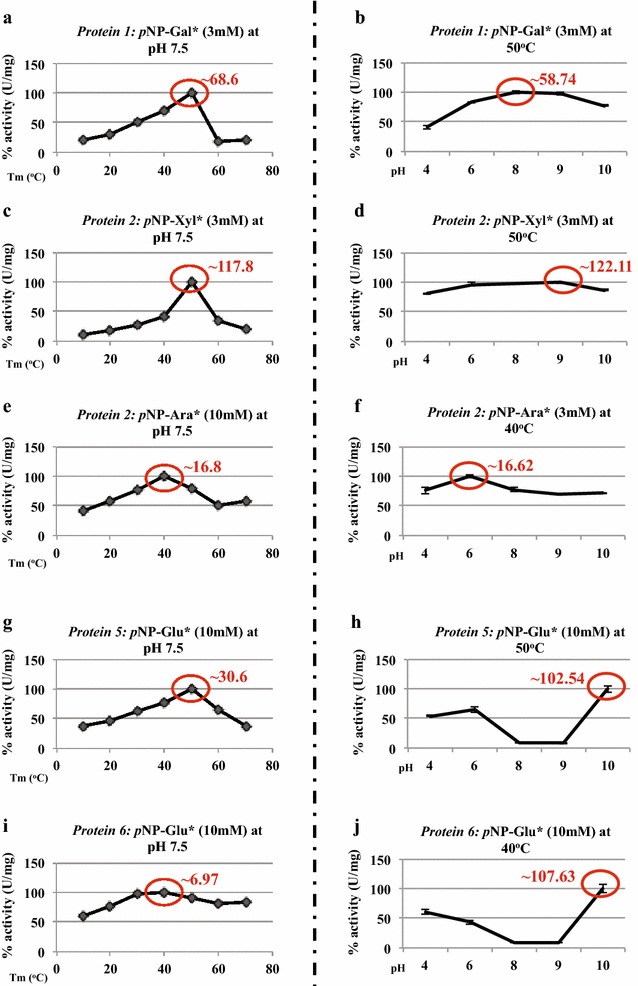
Characterization of four selected candidate GHases: Enzyme activities measured against *p*NP-substrates under different temperatures with constant pH and different pH with optimum temperature. *Protein 1* with *p*NP-β-D-galactopyranoside **a.** Tm 50°C; pH 7.5; 68.6 U/mg and **b.** Tm 50°C; pH 8.0; 58.74 U/mg; *Protein 2* with *p*NP-β-D-xylopyranoside **c.** Tm 50°C; pH 7.5; 117.8 U/mg and **d.** Tm 50°C; pH 9.0; 122.11 U/mg; *Protein 2* with *p*NP-α-L-arabinopyranoside **e.** Tm 40°C; pH 7.5; 16.8 U/mg and **f.** Tm 40°C; pH 6.0; 16.62 U/mg; *Protein 5* with *p*NP-α-D-glucopyranoside **g.** Tm 50°C; pH 7.5; 30.6 U/mg and **h.** Tm 50°C; pH 10.0; 102.54 U/mg; *Protein 6* with *p*NP-α Dglucopyranoside **i.** Tm 40°C; pH 7.5; 6.97 U/mg and **j.** Tm 40°C; pH 10.0; 107.63 U/mg

We scaled up the production of the gene 1, 2, 5 and 6 products in order to obtain sufficient soluble protein for further testing. The products were thus purified from large overnight *E. coli* Origami2 (DE3) pLysS cultures containing copies of intact genes 1, 2, 5 and 6 in the pET28b(+) expression vector (see Fig. 2). The products of genes 1 (~116 kDa), 2 (~85 kDa), 5 (~45 kDa) and 6 (~22 kDa) had the predicted molecular sizes, as estimated from the polyacrylamide gels. The purified proteins were then further examined for GHase activity under various conditions of temperature (10–70 °C) and pH (4.0–10.0), as discussed below and shown in Fig. 3.

#### Protein 1

The activity of the gene 1 encoded protein was examined with 3 mM *p*NP-Gal as the substrate. It was active over a temperature range from 30 to 50 °C (Fig. 3a), with maximal activity at 50 °C, of 68.6 U/mg. The effect of pH was then determined at 50 °C (Fig. 3b). Activity was observed in the pH range 4.0–10.0, with maximal activity at pH 8.0 (58.7 U/mg). This activity did not decrease much at pH 9.0 (98%) and 10.0 (78%).

#### Protein 2

Initially, the gene 2 encoded protein was screened for enzymatic activity on *p*NP-Xyl and *p*NP-Ara (both 3 mM), revealing both activities. The purified enzyme was found to be active on *p*NP-Xyl in the temperature range 40–60 °C (Fig. 3c), showing highest activity at 50 °C, with the release of approximately 117.8 U/mg of *p*-nitrophenol. As in Fig. 3d, activity was further observed between pH 6.0 and 10.0, with maximal activity at pH 9.0, at 50 °C. The activity, overall, remained >60% between pH 4.0 and 10.0. On *p*NP-Ara, the enzyme showed activity (~16.8 U/mg) between 20 and 70 °C, with maximal activity at 40 °C (Fig. 3e). We then checked the pH sensitivity of the enzyme using *p*NP-Ara at 40 °C, and found optimal activity at pH 6.0, with approximately 16.62 U/mg of *p*-nitrophenol being released (Fig. 3f).

#### Protein 5

To define the optimal temperature for the activity testing of protein 5, tests were performed with *p*NP-α-d-glucopyranoside (10 mM) at pH 7.5 and at temperatures ranging from 10 to 70 °C. An optimal temperature of 50 °C for protein 5 activity was found (Fig. 3g). Moreover, the protein also released 30.6 U/mg of *p*-nitrophenol. The latter activity (at 50 °C) was maximal at pH 10.0 (102.54 U/mg; Fig. 3h).

#### Protein 6

The gene 6 product was tested with 10 mM of *p*NP-α-d-glucopyranoside at pH 7.5 and in a temperature range of 10 to 70 °C. Protein 6 worked optimally at 40 °C, with 6.97 U/mg of *p*-nitrophenol being released (Fig. 3i). Furthermore, the enzyme showed maximal activity, i.e., 107.63 U/mg of *p*-nitrophenol being released, at pH 10.0 (Fig. 3j), and this was slightly higher than the product of gene 5.

#### Comparison of gene 5 and gene 6 products

The products of genes 5 and 6 showed similar enzymatic activities on *p*NP-α-d-glucopyranoside at pH 4.0–6.0 under the same conditions. Unexpectedly, the activities of both proteins 5 and 6 dropped to near zero at pH 8.0–9.0 (Fig. 3h, j). Moreover, protein 5 showed significantly higher α-glucosidase activity (30.6 ± 0.17 U/mg) than protein 6 (6.9 ± 0.36 U/mg).  The collective results suggest that the four selected genes encode proteins with different thermo-alkaliphilic activities, as they all were optimally functional at temperatures of 40–50 °C and pH values of up to 10.0 (Fig. 3).

The kinetic parameters of all enzymes were calculated from Lineweaver–Burk plots of specific activities at various substrate concentrations (0–10 mM). The *K*_m_ and *V*_max_ values for protein 1 (with *p*NP-β-d-galactopyranoside) were 0.1 mM and 58.8 U/mg and for protein 2 (*p*NP-β-d-xylopyranoside and *pNP*-α-l-arabinopyranoside) 1.0 and 0.3 mM, and 666.7 and 102 U/mg, respectively. The *K*_m_ values of proteins 5 and 6, with *p*NP-α-d-glucopyranoside, were 7.4 and 21.4 mM, and the *V*_max_ values were 196 and 588.2 U/mg, respectively.

### Stability of the selected enzymes at elevated temperatures

**Fig. 4:**
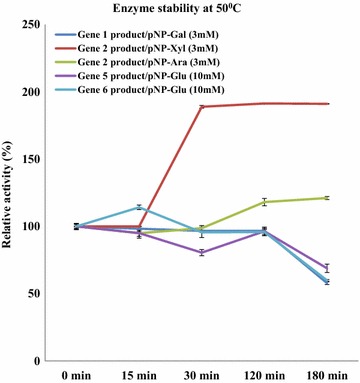
The effect of temperature on enzyme stabilities. Explanation: activities of proteins 1, 5 and 6 were stable up to 120 min (at 50 °C); protein 2 showed increased activity up to 180 min at 50 °C

In a second set of experiments, we determined the stabilities of the enzymes encoded by genes 1, 2, 5 and 6, at elevated temperatures. The gene 1 (*p*NP-Gal), 5 (*p*NP-Glu) and 6 (*p*NP-Glu) products retained 95% of activity after over 120 min of incubation at 50 °C, after which the activities decreased—at 180 min—to about 50% of the initial levels. At 50 °C, the gene 2 product showed 100% activity with *p*NP-Xyl for 15 min, and then enzyme activity even increased, to over 180% of the control, maintaining the raised activity level until 180 min. On *p*NP-Ara, the gene 2 encoded protein retained 100% activity for 30 min, showing a 20% increased activity in the period between 120 and 180 min (Fig. 4).

### Effects of inhibitors (5-HMF and furfural) and degradation of complex polysaccharide

**Fig. 5:**
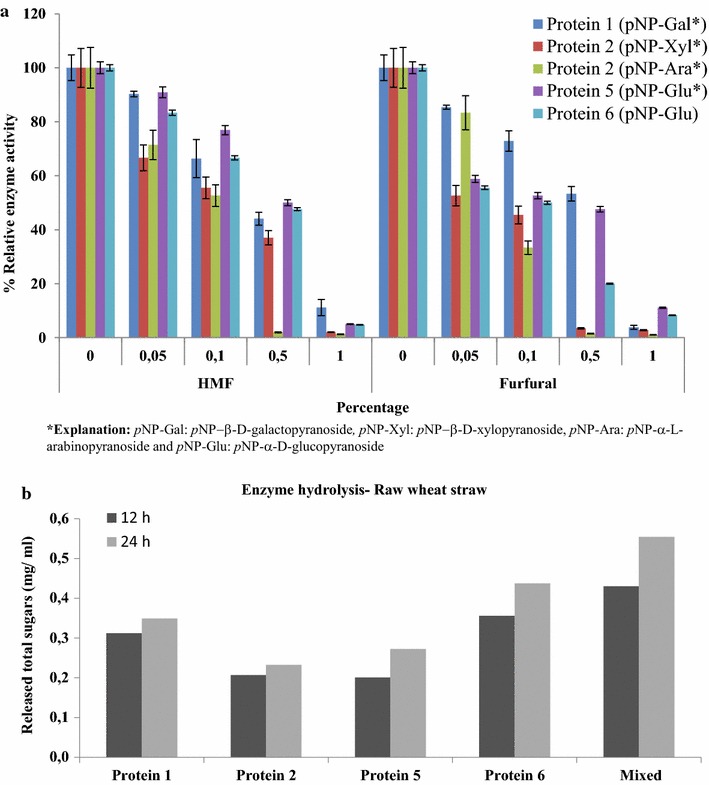
Relative activities of proteins 1, 2, 5 and 6. **a.** different concentrations (zero to 1.0% (w/v)) of furfural and 5-hydroxymethylfurfural (5-HMF); **b.** effect of lignocellulosic polysaccharide hydrolysis (raw wheat straw)

Two of the major inhibitors in lignocellulose hydrolysis, 5-hydroxymethyl furfural (HMF) and furfural were tested for their effects on the activity proteins 1, 2, 5 and 6 using selected concentrations of *p*NP substrates (3 mM of *p*NP-Gal, *p*NP-Xyl and *p*NP-Ara; 10 mM of *p*NP-Glu) (Fig. 5a). The activities of all enzymes were in most cases strongly blocked in the presence of 1.0% (w/v) 5-HMF and furfural (approximately 90–95%). Interestingly, the presence of 0.5% (w/v) 5-HMF inhibited all enzymes by 50-60%, whereas protein 2 with *p*NP-Ara showed nearly 100% inhibition. At the lower dosages (0.05–0.1% w/v), 5-HMF inhibited all enzymes by 10–40% (Fig. 5a). Next to that, the presence of 0.5% (w/v) furfural resulted in an inhibitory effect of 50% to proteins 1 and 5, but in one close to 100% for protein 2 (with *p*NP-Xyl and *p*NP-Ara) (Fig. 5a). Notably, low levels (0.05–0.1% w/v) of furfural showed about 10–70% inhibition.

The efficiency of degradation of raw wheat straw by the four enzymes was determined by measuring the total sugars released. The data suggested that the 24 h treatment showed higher hydrolysis effect for all enzymes (proteins 1, 2, 5 and 6) when compared with the 12 h treatment (Fig. 5b). The amount of total sugars was higher in the presence of protein 6 (0.44 mg/ml) when compared with the other three proteins. Moreover, the addition of protein 1 yielded 0.35 mg/ml of total sugar. Interestingly, addition of all enzymes together (mixed) increased the 24 h yield up to 0.55 mg/ml (Fig. 5b).

### Enzyme activities in the presence of ions

To examine the effects of ions (including NaCl) on the four selected enzymes (Table 2), activity tests under different ionic regimes were carried out with the appropriate substrates. In the presence of all MgCl_2_, MnCl_2_ and NaCl levels, the enzymes encoded by genes 1, 2 and 6 showed increased activities, of between 10 and 25% of the control (without ions). Protein 2, on *p*NP-Ara, showed significantly increased (*P* < 0.05) activity (50%) in the presence of 5 mM of Mg^2+^, as compared to the controls without ions, and almost 80% elevated activity with 200 mM of NaCl. Finally, protein 6 showed increased α-glucosidase activity (50%) in the presence of 200 mM of NaCl when compared to the controls without NaCl. In fact, all additives, except 5 mM of Mg^2+^, significantly increased this enzymatic activity. Strikingly, the protein 5 α-glucosidase activity decreased in the presence of all additives; the activity was significantly lowered upon addition of both 5 mM of Mg^2+^ and 2000 mM NaCl (p<0.05).

**Table 2:** Effects of metal ions and NaCl on the enzymatic activities of recombinant enzymes (each treatment had three biological replicates) ^a^Substrate: refer to Fig. 5a

Enzymes (product of:)	Substrate^a^	No additive	Relative activities (%)					
			MgCl_2_		MnCl_2_		NaCl	
			5 mM	20 mM	5 mM	20 mM	200 mM	2000 mM
Gene 1	*p*NP-Gal	100 ± 1.8	113.1 ± 9.1	114.1 ± 9.6	97.7 ± 6.5	95.5 ± 9.2	91.7 ± 3.5	90.9 ± 3.6
Gene 2	*p*NP-Xyl	100 ± 4.9	124.5 ± 0.7	129.9 ± 4.9	108.6 ± 4.4	105.0 ± 3.2	112.4 ± 12.4	118.6 ± 9.3
Gene 2	*p*NP-Ara	100 ± 17.6	150.5 ± 19.4	106.4 ± 26.0	135.5 ± 7.6	81.6 ± 1.2	183.9 ± 53.1	75.1 ± 1.9
Gene 5	*p*NP-Glu	100 ± 2.7	85.4 ± 3.4	94.0 ± 6.3	95.6 ± 2.3	88.7 ± 1.0	102.7 ± 3.8	59.9 ± 2.4
Gene 6	*p*NP-Glu	100 ± 4.1	105.5 ± 1.4	112.8 ± 5.5	92.8 ± 2.3	111.1 ± 1.0	159.5 ± 3.6	119.3 ± 5.1

### Analysis of the sequences of proteins 5 and 6

#### Evidence for a new α-glucosidase encoded by gene 5

**Fig. 6:**
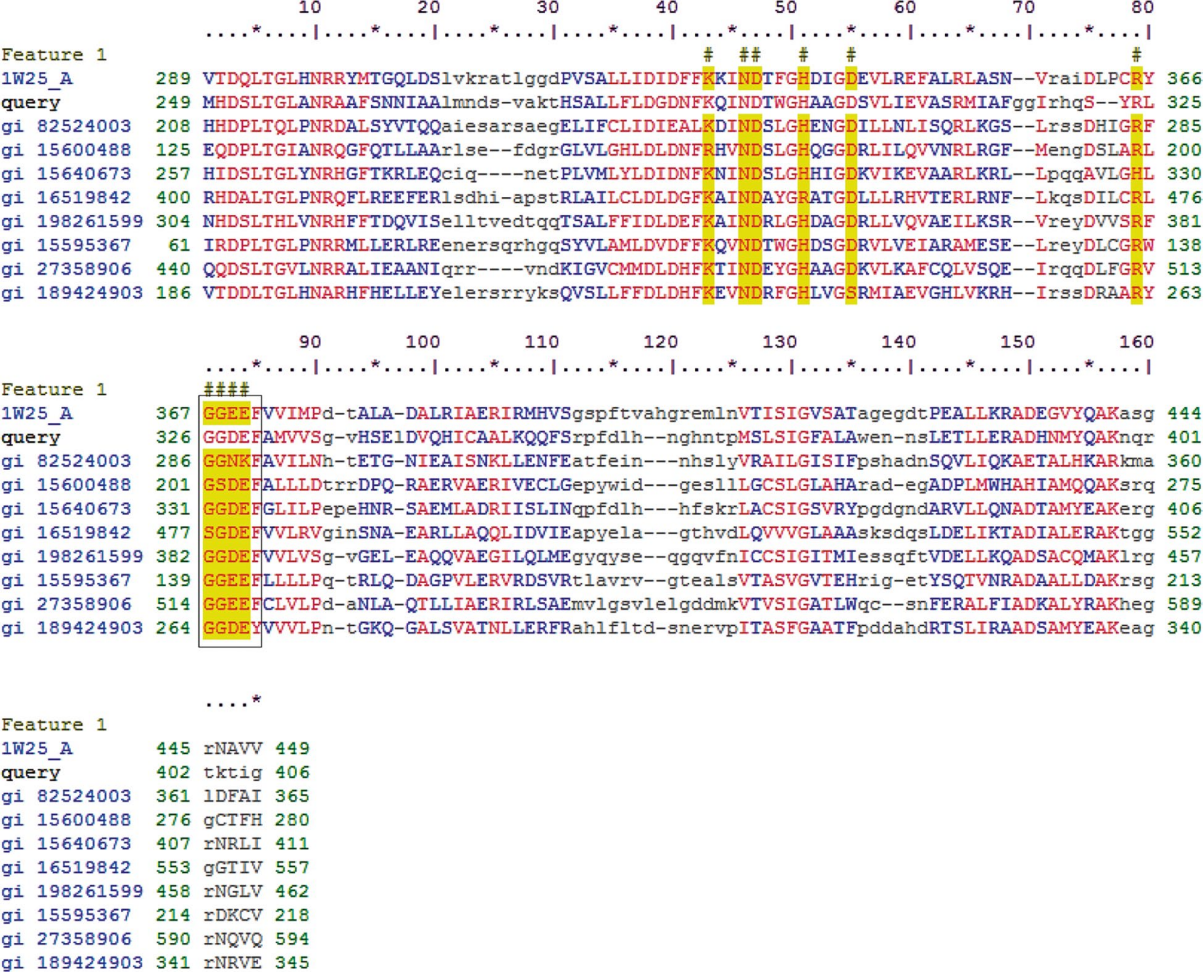
Alignment of amino acid sequences revealing that protein 5 contains the (typical for class III nucleotidyl cyclases [37]) conserved residues K 290: lysine, N 293: asparagine, D 294: aspartic acid, H 298: histidine, D 302: aspartic acid, and a “GGDEF” domain (R 324: arginine, G 326: glycine, G 327: glycine, D 328: aspartic acid, E 329: glutamic acid and F 330: phenylalanine). 1W25_A-diguanylate cyclase [*Caulobacter vibrioides*]; gi 82524003-Hypothetical protein [uncultured gamma *proteobacterium*]; gi 15600488-hypothetical protein PA5295 [*Pseudomonas aeruginosa* PAO1]; gi 15640673- c-di-GMP phosphodiesterase A-like protein [*Vibrio cholerae*]; gi 16519842-diguanylate cyclase/phosphodiesterase [*Sinorhizobium fredii *NGR234]; gi 198261599-Sensory box/ggdef domain/eal domain protein [gamma *proteobacterium* HTCC5015]; gi 15595367-Hypothetical protein PA0169 [*Pseudomonas aeruginosa* PAO1]; gi 27358906-FOG: GGDEF domain protein [*Vibrio vulnificus* CMCP6]; gi 189424903-GAF sensor-containing diguanylate cyclase [*Geobacter lovleyi* SZ]

Analyses of protein 5, using BLAST-P, multiple sequence alignment and subsequent tree building with the first 19 matches, revealed between 81 and 92% identity of the protein with proteins defined as ‘diguanylate cyclase’ from, respectively, *Kluyvera cryocrescens* and *Enterobacter cloacae* (Additional file 1: Figure S1). Further characterization of the domains of protein 5 using Pfam revealed a quite complex domain architecture. Two putative conserved domains were identified, denoted as ‘HAMP’ and ‘GGDEF’ (Fig. 6). The HAMP domain has been described as a signaling mediator, being mostly found in histidine kinases, adenyl cyclases, methyl-accepting proteins and phosphatases [23]. HAMP domains consist of 16-residue amphiphilic helices that are often part of a two-component signal transduction pathway [24]. They can be found in association with other domains, such as the GGDEF and EAL domains. Remarkably, gene 5 is here described as a gene encoding a glycoside hydrolase family GH53 protein (analysis by the CAZymes analysis toolkit (CAT) server), with predicted endo-β-1, 4-galactanase activity based on the (catalytic) GGDEF domain. Therefore, we here posit that protein 5 is a GGDEF family protein, which is part of a signal-responsive system (given its HAMP domain), with α-glucosidase activity as shown with *p*NP-α-d-glucopyranoside.

Furthermore, we analyzed the protein 5 amino acid sequence based on domain architecture and revealed potential “GDSL” motifs (by CDART); the presence of such a GDSL motif may indicate the potential for multiple functional properties, such as broad substrate specificity, given its active site flexibility. Due to this, multiple activities, such as lipase and esterase activities, could be present, which are of use in hydrolysis processes of biological interest [25]. Further 3D modeling of protein 5 (Fig. 8a) indicated the presence of hypothetical ligands of both the GGDEF and GDSL domains that may allow binding of a particular substrate, supporting a catalytic mechanism.

#### Evidence for a new α-glucosidase encoded by gene 6

**Fig. 7:**
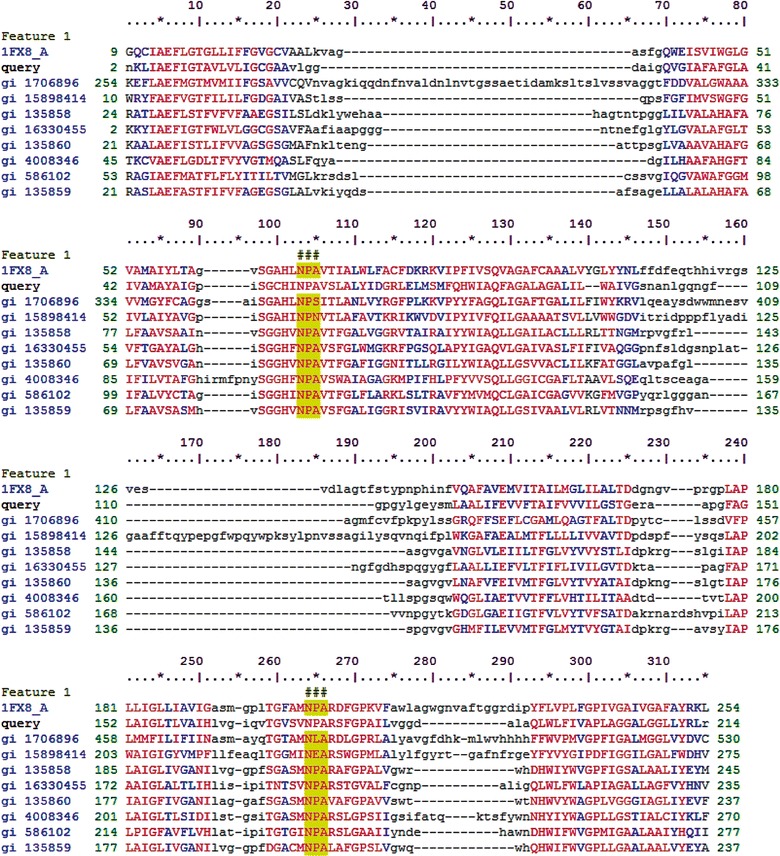
Alignment of protein 6 amino acid sequences showed two highly conserved NPA motifs (asparagine–proline–alanine; highlighted yellow). 1FX8_A-Membrane protein [*Escherichia coli*]; gi 1706896-Glycerol uptake/efflux facilitator protein [*Saccharomyces cerevisiae* S288C]; gi 15898414-transposon ISC1229 Orf1 [*Sulfolobus solfataricus* P2]; gi 135858-Aquaporin TIP3-1 [*Arabidopsis thaliana*]; gi 16330455-Aquaporin Z [*Synechocystis* sp. PCC 6803]; gi 135860-Aquaporin TIP1-1 [*Arabidopsis thaliana*]; gi 4008346-Major intrinsic protein [*Caenorhabditis elegans*]; gi 586102-Membrane protein [*Solanum lycopersicum*]; gi 135859-Aquaporin TIP-type alpha [*Phaseolus vulgaris*]

**Fig. 8:**
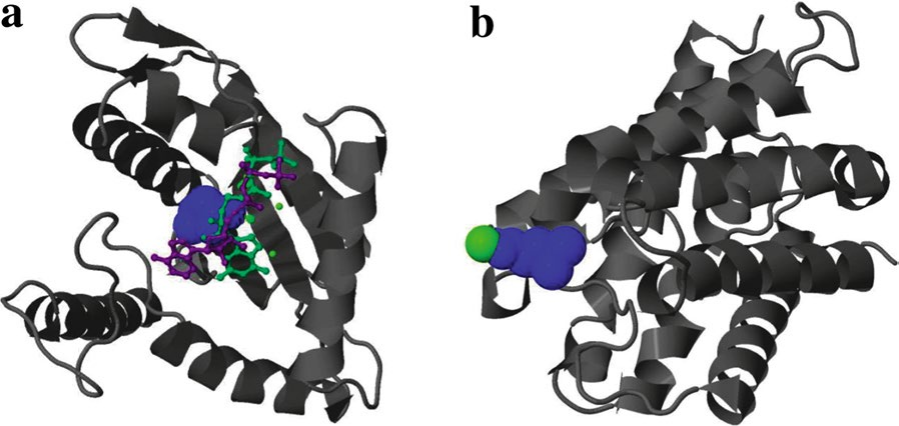
Three-dimensional (3D) models of **a.** protein 5 and **b.** protein 6. Background helices shown in *gray*, Active sites shown in *blue*, Hypothetical ligands shown in *magenta* and *green*

In the case of the gene 6 product, a BLAST-P search initially showed high similarity of it to typical porin-like proteins, with up to 97% identity. Specifically, the predicted protein clustered with porins from the genera *Martelella endophytica* (80%) and *Devosia* sp. *root635* (97%), which are both Alphaproteobacteria from the order Rhizobiales (Additional file 2: Figure S2). Connected to this, the characterization of the different domains in the predicted protein yielded a match with transmembrane channel-forming proteins of the major intrinsic protein (MIP) family from *Hyphomonas johnsonii* and *Brevundimonas diminuta*. The predicted protein 6 was found to contain the characteristic Asn-Pro-Ala (denoted as NPA) signature motif located at the segment interface between helices M3 and M7 in residues 103–105 (Fig. 7, in yellow). In our previous study, the source fosmid clone NT18-17 produced proteins with α-glucosidase activity. Here, the product of the cloned gene 6 showed 100% increased α-glucosidase activity at pH 10.0 (Fig. 3j). Finally, protein 6 was analyzed with respect to domain architecture. A potential “GDSL” motif was also found by the CDART software, the potential function, and characterization of this motif being as above. Using 3D modeling (Fig. 8b), hypothetical substrate-binding ligands were identified, supporting the tenet that a catalytic site is present in this presumed transmembrane protein.

## Discussion

Metagenomics constitutes a true ‘breakthrough tool’ that allows to examine natural and manipulated microbiomes for the presence of genes for novel enzymes that may fuel biomedical and industrial applications [26]. In a previous study involving two wheat straw-degrading microbial consortia, we identified two genes encoding proteins with putative thermo-alkaliphilic activities [14]. With respect to activity (β-galactosidase and β-xylosidase), these were stable at alkaline pH and elevated temperature. Hence, they may have interesting potential applications in the process of enzyme-assisted pulp bleaching [14]. In the current study, we further mined our fosmids that were predicted to encode such GHases. Eight thus selected genes were cloned into two *E. coli* host strains, BL21(DE3) and Origami2 (DE3) pLysS, and gene expression was examined. Strain BL21(DE3) is useful for gene expression studies from a T7-type promoter on the vector. In our study, BL21(DE3) cell growth was arrested, possibly as a result of the emergence of inhibitory compounds, i.e., the intended proteins, in the culture. In the case of *E. coli* Origami2 (DE3) pLysS, the T7 lysozyme produced suppresses the basal expression of T7 RNA polymerase prior to induction. Thus, we found *E. coli* Origami2 (DE3) pLysS to produce more proteins in the soluble fractions than BL21(DE3), giving rise to five (genes 1, 2, 3, 5 and 6) measurable enzyme activities. In our previous work [14], fosmid clone 10BT extracts showed activity with multiple substrates and also light (14.8%) β-xylosidase activity. There was no detectable α-glucosidase activity. Interestingly, protein 5 (originating from clone 10BT) showed α-glucosidase activity from the strong inducible promoter of the vector. Thus, presumably the native promoter of this gene did not allow for expression under such conditions. Concerning genes 7 and 8, no activity was found with any of the test substrates, of which we ignore the cause. However, problems of instability and/or aggregation (under the prevailing conditions of osmolarity, pH, redox potential, cofactors) may have played key roles.

Based on the initial screening, we thus considered the selected five genes for further study of their products. Among these, proteins 2 and 3 were similar in activity and size. With reference to our previous study, here we selected gene 2 instead of gene 3 (from fosmid clone T5-5) based on the high thermo-alkaliphilic activity with *p*NP-Xyl, whereas the gene 3 mother fosmid clone T4-1 had shown only slight activity against *p*NP-Xyl [14].

### Protein 1

With respect to the identification of protein 1 as an EC. 3.2.1.23 (GH2), several new β-galactosidase family -GH2 enzymes have recently been discovered and characterized [27–29]. Specifically, a purified (β-galactosidase) family -GH2 enzyme, denoted BglA, from *Arthrobacter psychrolactophilus F2* showed maximal enzymatic activity (alkaliphilic) at pH 8.0/10 °C (33.3 U/mg). Such β-galactosidases are widely used in the food industry given their capabilities to hydrolyze lactose at extreme (low or high) temperatures, and to produce glucose and galactose. Remarkably, our gene 1 encoded protein showed increased enzymatic activity at raised temperature and similar pH when compared to the BglA enzyme, with increased *p*-nitrophenol release (58.7 U/mg; Table 3). Moreover, protein 1 showed a lower *K*_m_ and slightly increased *V*_max_ as compared to those of the aforementioned *A. psychrolactophilus* enzyme, as well as a and *Lactobacillus sakei* Lb790 enzyme [27, 29]. Therefore, protein 1 shows promising hydrolytic activity, which may be of direct relevance to industrial processes.

**Table 3:** Comparison with enzymes (GH2 and GH3/43) from other studies (reactions with the substrate ONPG and/or PNPG)

Enzyme family	Functions	Strain	Protein (kDa)	Optimum Tm (°C)	Optimum pH	Activity (U/mg)	References
GH 2	β-Galactosidase	*Arthrobacter psychrolactophilus F2*	130	10	8	33.3	Nakagawa et al. [27]
GH 2	β-Galactosidase	*Arthrobacter* sp. *20B*	113.7	25	6–8	0.84	Bialkowska et al. [56]
GH 2	β-Galactosidase	*Arthrobacter* *SB*	114	18	7	26.9	Coker and Brenchley [57]
GH 2	β-Galactosidase	*Flavobacterium* sp. *4214*	114.3	42	7.5	–	Sørensen et al. [58]
GH 2	β-Galactosidase	*Halomonas* sp. *S62*	63	45	7	–	Wang et al. [59]
GH 2	β-Galactosidase	*Enterobacter hormaechei*	120	50	8	58.7	This study
GH 3	Dual function (β-xylosidase/α-arabinofuranosidase)	*Rumen/Parabacteroides distasonis ATCC 8503*	80	50	6	29.5	Zhou et al. [30]
GH 43	Dual function (β-xylosidase/α-arabinofuranosidase)	*Rumen/Prevotella bryantii*	45	40	7	36.3/14.2	Zhou et al. [31]
GH3–BglX	Dual function (β-xylosidase/α-arabinofuranosidase)	*C. crescentus*	90	60/50	6	4.3/3.0	Justo et al. [32]
GH 3	Dual function (β-xylosidase/α-arabinofuranosidase)	*Enterobacter mori*	80	50/40	9 and 6	122.11/16.6	This study

### Protein 2

Gene 2 was annotated as a gene encoding a protein with β-xylosidase/α-arabinosidase activity belonging to CAZy family GH3 (EC. 3.2.1.37). As shown in Table 3, the enzyme had raised activities on *p*NP-Xyl and *p*NP-Ara, with optimum pH values of 9.0 (and 6.0) at temperatures of 50 and 40 °C. This was superior as compared to the previously reported enzymes Rubgx1, GH43 (several) and XynB5. Specifically, the Rubgx1 (GH3 family) enzyme (β-glucosidase/β-xylosidase activities; optima at pH 6.0 and 50 °C) [30], the GH43 family one (β-xylosidase/α-arabinosidase; optima at pH 7.0 and 40 °C) [31] and, finally, XynB5 (β-glucosidase/β-xylosidase/α-arabinosidase; optimum pH 6.0 and temperature 50 °C) had lower activities than protein 2 [32]. In addition, protein 2 revealed high kinetic parameters, such as *V*_max_ (666.6 and 102 U/mg), than a previously-characterized bifunctional (β-xylosidase/α-arabinosidase) enzyme [31]. In another study, we recently characterized GH43 family enzyme XylM1989 with β-xylosidase/α-arabinosidase activity and showed *V*_max_ values of 285.71/78.12 U/mg, respectively (unpublished data). Also, protein 2 progressively showed higher enzyme activity (with the corresponding substrates) with increasing pH. Such changes in pH will alter the attractions between groups in the side chains of the protein, potentially modifying the protein domain shape. Moreover, the binding of substrate to the active site may be modulated and/or it cannot undergo catalysis.

As indicated above, protein 2 was similar to the GH3 family proteins Rubgx1 (81.4 kDa) and XynB5 (95 kDa, from *Caulobacter crescentus*) [30, 32]. Its *p*NP-Xyl and *p*NP-Ara dual activity may have been due to two distinct catalytic properties in the same polypeptide chain, usually catalyzing two consecutive reactions [33]. Dual- or even multi-functional properties are common in proteins of the GH3 (and GH43) family. For instance, the GH3 family protein XynB5 showed rather similar β-glucosidase/β-xylosidase/α-arabinosidase activities [32]. Moreover, GH3 family enzymes include β-xylosidases (EC 3.2.1.37) and α-arabinosidases (EC 3.2.1.55). Whereas the aforementioned enzymes Rubgx1 and XynB5 showed (β-xylosidase) activities with optimum pH and temperature of 6.0 and 50 °C, respectively, protein 2 showed maximal activity at pH 9.0 and 50 °C (122.11 U/mg) (Fig. 3d). This preference for alkaline instead of slightly acidic conditions is likely connected to amino acid substitutions that we have—as yet—not addressed. The mechanisms by which an enzyme’s catalytic properties may be affected were beyond the scope of the current study [34].

### Protein 5

Interestingly, protein 5 showed α-glucosidase activity, whereas no such activity was detected from its mother fosmid clone 10BT [14]. Fosmid clone 10BT had shown activity in the presence of multiple substrates, revealing some activity with *p*NP-Xyl. Thus, expression of the α-glucosidase encoding gene may have been repressed at the genomic level, becoming expressed from the inducible promoter of the expression system of the used vector. Protein 5 clearly belongs to the “GGDEF” domain protein family [35, 36]. This domain was first identified in a response regulator involved in cell differentiation in *C. crescentus* [37]. It was also observed in *Salmonella enterica* enzymes involved in cellulose biosynthesis and biofilm formation [38]. For more than 20 years, all GGDEF domain enzymes have been classed as diguanylate cyclases and/or phosphodiesterases [39]. The former enzymes produce cyclic di-GMP (cdiG), a messenger that regulates the key bacterial lifestyle transition from a motile to a sessile, biofilm-forming, state [40]. However, most bacteria are known to possess large numbers of genes that encode a range of GGDEF domain proteins [38], allowing functional diversity across them. The function of most GGDEF domain containing proteins has not yet been experimentally proven [41]. Surprisingly, the GGDEF domain family protein 5 revealed α-glucosidase activity (in the context of (hemi)cellulose degradation), with a 100% raised activity at pH 10.0 (102.54 U/mg) as compared to pH 7.0. At pH 10, the large OH^−^ oversupply may have caused a change in the shape and/or charge of the enzyme’s active site, spurring activity. Further detailed structural studies could reveal the dynamics of such glycoside hydrolase activities at varying pH values in the lignocellulose degradation processes. Also, protein 5 was found to have a different motif, of the “GDSL” class by CDART prediction.

### Protein 6

Remarkably, protein 6 was found to possess two different conserved motifs, NPA and GDSL. The NPA motif is a key structural feature of proteins that plays crucial roles as water channels across membranes, supporting membrane localization of the protein. Surprisingly, protein 6 also belongs to the GDSL hydrolase family (encompassing esterases/lipases), not sharing sequence homology with any of the CAZy database glycosyl hydrolases. GDSL motif enzymes constitute a rather new group of proteins, with characteristics that have not yet been precisely described [42]. Furthermore, a subgroup of this GDSL family was categorized as a so-called SGNH hydrolase, and our protein 6 belongs to this subgroup. The SGNH family of hydrolytic enzymes has a wide range of catalytic functions, such as lipase, protease, carbohydrate esterase, (thio)esterase, arylesterase and acyltransferase activities [43]. All GDSL hydrolase family enzymes were found to have flexible substrate-binding and/or active sites [43]. Indeed, the Koshland induced-fit theory indicates that the active sites of enzymes may become modified in the presence of substrates, involving structural and catalytic site modifications [44]. The active and/or binding sites of protein 6 are presumably flexible if they are to follow the induced-fit theory. For instance, the newly-identified carbohydrate esterase family 3 (CE3) gene *axe2* product (acetyl xylan esterase), which has a GDSL motif, removes acetyl groups from the hemicellulose polymer xylan [45]. Such activity, i.e. carbohydrate esterase, likely gives protein 6 the capability to hydrolyze substituents on the xylan backbone, supporting its ability to competently degrade hemicellulose [46]. Indeed, protein 6 (and 5), based on an SGNH-type hydrolase, may exhibit the specific α-glucosidase activity in (hemi)cellulose degradation.

In recent years, several enzyme cocktails have been proposed that can enhance plant biomass degradation rates [47]. Such cocktails were produced from several sources, including the producer organisms *Trichoderma reesei, Thermobifida fusca* and *Clostridium thermocellum*. The degradation of lignocellulose requires the intensive action of multiple enzymes. This is due to the structural aspects that are typical for the substrate, i.e., its high molecular weight and the association of hemicellulose with cellulose and lignin. The required hemicellulases need to hydrolyze glycosidic bonds. In many habitats, the degradation process is rather slow because of the poor substrate accessibility, inhibitors (5-HMF and furfural), and untimely presence of efficient enzymes. However, in industrial processes, such enzyme availabilities can be steered [48, 49]. The inhibitory by-products 5-HMF and furfural are released during lignocellulose degradation, and constitute a major inhibitors of subsequent fermentation [50, 51]. However, the amount of 5-HMF and furfural in plant biomass hydrolysates is strongly linked to the pretreatment methods and the source of the plant biomass. In this line, the levels of 5-HMF and furfural in corn, poplar and pine were 0.017 and 0.022% (w/v), respectively [52]. Interestingly, the four enzymes (proteins 1, 2, 5 and 6) showed 50–80% tolerance to 0.1% 5-HMF, and about 40–50% tolerance to 0.5% (w/v). In addition, all enzymes also revealed tolerance (30–72%) to furfural. Remarkably, protein 5 (with α-glucosidase activity) revealed 50% activity at 0.5% of 5-HMF and furfural. Therefore, we posit that such inhibitor-tolerant galactosidases and glucosidases have great interest in food and biorefinery industries. Because of this strong tolerance, our enzymes may work better than other ones in the presence of certain levels of inhibitors. As of now, no previous studies reported 5-HMF- and furfural-tolerant β-galactosidases and α-glucosidases.

The four (thermo-stable) enzymes described here may pave the way towards improved (hemi) cellulolytic action of existing enzyme cocktails. Using the his-tags, the enzymes, provided they are sufficiently stable, can be easily recovered from the treatment process and re-used in continuous applications, allowing alkaline and enhanced temperature conditions [53]. The here-detected activity of our enzymes (specifically proteins 1 and 2) under thermo-alkaliphilic conditions thus indicates their usefulness for plant biomass degradation, spurring the production of glucose and xylose/arabinose from (hemi)cellulosic material and also for pulp bleaching processes. Moreover, thermo-stable enzymes like the ones reported here are often specific for activity on particular bonds and therefore offer promise for specific industrial purposes [54, 55]. Several amylases and cellulases are already in use in industries, including enzymes with wide pH range [4.0–10.0] and high thermo-tolerance. They are clearly useful under conditions that restrict microbial growth. Furthermore, enzymes with optimized properties, such as enhanced thermal and alkali tolerance, and an ability to function without additives, recently improved several industrial applications [51, 52]. In a subsequent study, we will characterize the role of the novel enzymes as modulators of the degradation of hemicellulose compounds.

## Additional files

**Additional file 1: Figure S1.** Protein 5 Blast-P multiple sequence alignment.

**Additional file 2: Figure S2.** Protein 6 Blast-P multiple sequence alignment.

## Abbreviations

CAZy: carbohydrate-active enzymes database
GHase: glycosyl hydrolases
ORF: open reading frame
*p*NP: *para*-nitrophenyl
RAST: Rapid Annotations using Subsystems Technology
*p*NP-Gal: *p*NP-β-d-galactopyranoside
*p*NP-Xyl: *p*NP-β-d-xylopyranoside
*p*NP-Ara: *p*NP-α-l-arabinopyranoside
*p*NP-Glu: *pNP*-α-d-glucopyranoside
CBMs: carbohydrate-binding modules
AAs: auxiliary activities
GTs: glycosyl transferases
CEs: carbohydrate esterases
