# Supplementary figures and images for: Molecular cloning, expression, and characterization of four novel thermo-alkaliphilic enzymes retrieved from a metagenomic library
Source: Biotechnol Biofuels. 2017 Jun 2;10:142. doi: 10.1186/s13068-017-0808-y (PMC5457731; doi:10.1186/s13068-017-0808-y)

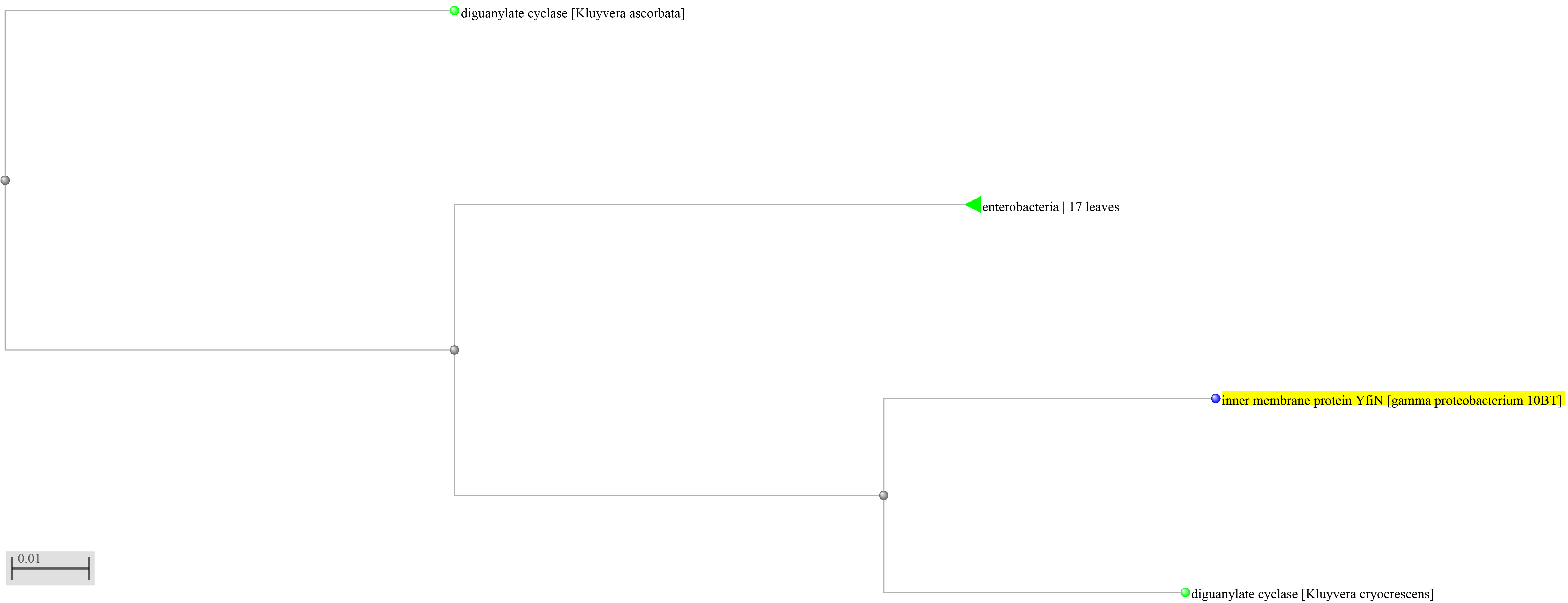

Supplement: Supplementary file 1 — Additional file 1: Figure S1. Protein 5 Blast-P multiple sequence alignment. [file 13068_2017_808_MOESM1_ESM.jpg]

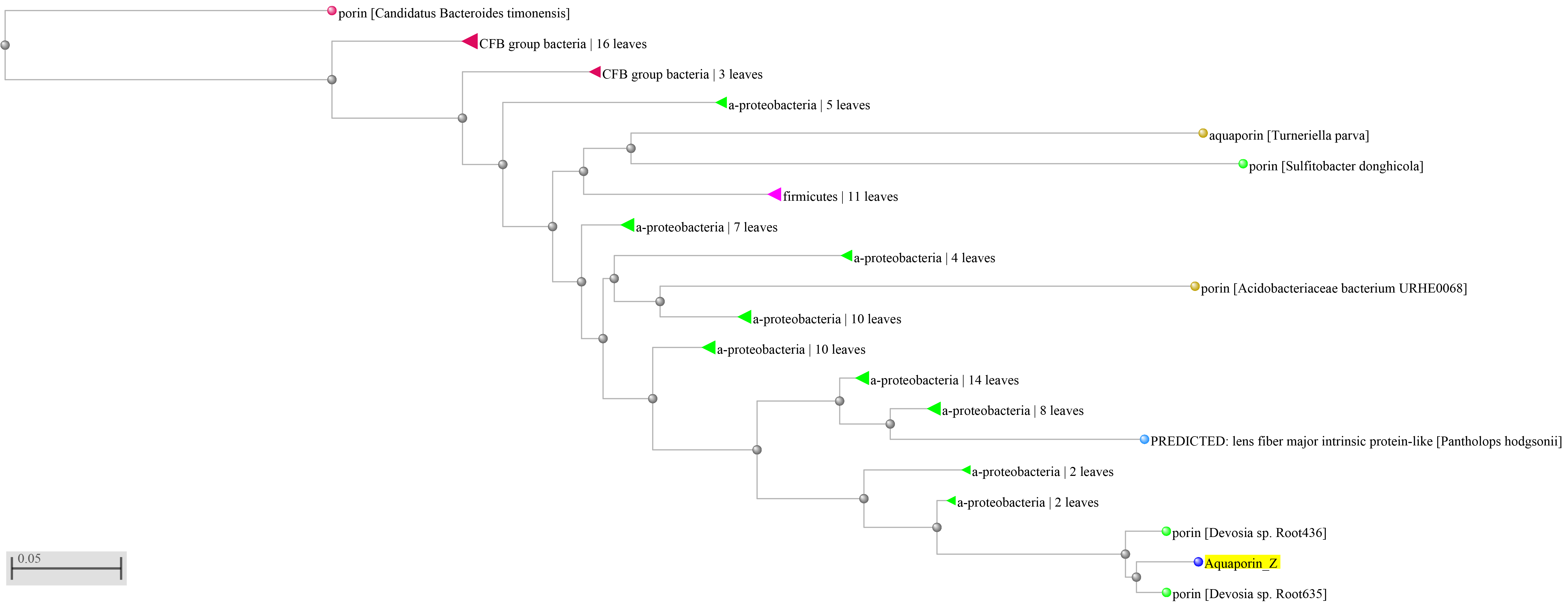

Supplement: Supplementary file 2 — Additional file 2: Figure S2. Protein 6 Blast-P multiple sequence alignment. [file 13068_2017_808_MOESM2_ESM.jpg]
